# Rationale and study design of a randomized controlled trial to investigate the renoprotective effect of canagliflozin assessed by test of renal hemodynamics in diabetic kidney disease (the FAGOTTO study)

**DOI:** 10.1186/s12882-023-03277-0

**Published:** 2023-08-03

**Authors:** Sawako Kato, Yachiyo Kuwatsuka, Masahiko Ando, Yoshitaka Tatematsu, Nobuhiro Nishibori, Shoichi Maruyama

**Affiliations:** 1 Department of Nephrology, Nagoya University Graduate School of Medicine, 65, Tsuruma-Cho, Showa-Ku, Nagoya, Aichi 464-8550 Japan; 2 Department of Advanced Medicine, Nagoya University Hospital, Aichi, Japan

**Keywords:** Canagliflozin, Clinical trial, Diabetic kidney disease, Renal hemodynamics, Renoprotection

## Abstract

**Background:**

Sodium-glucose cotransporter 2 inhibitors (SGLT2i) are considered to have the potential to maintain renal function by correcting glomerular hypertension in patients with diabetic kidney disease (DKD). The aim of this study is to demonstrate the renoprotective effect of SGLT2i by measuring renal hemodynamics, including glomerular filtration fraction (FF), in type 2 diabetic patients with moderate renal dysfunction.

**Methods:**

Renoprotective effect of canagliflozin derived from test of renal hemodynamics in diabetic kidney disease (FAGOTTO) study is a 12-week multicenter, open-label, randomized (1:1), parallel-group trial of type 2 diabetic patients with diabetic kidney disease (30 ≤ estimated glomerular filtration rate [eGFR] ≤ 60 mL/min/1.73 m^2^). A total of 110 patients are to be randomly allocated to receive once-daily canagliflozin 100 mg or control (standard therapy). FF will be calculated by dividing the measured GFR (mGFR) by the effective renal plasma flow (eRPF). mGFR and eRPF will be measured by the clearance of inulin and para-aminohippuric acid (PAH), respectively. The primary endpoint of this trial is the percentage change in FF after 4 weeks of treatment in the canagliflozin and control groups.

**Discussion:**

The FAGOTTO study will elucidate the mechanism of the renoprotective action of SGLT2i. The background, rationale, and study design of this trial are presented. To date, > 80 patients have been enrolled in this trial. The study will end in 2025.

**Trial registration:**

jRCT (Japan Registry Of Clinical Trials) jRCTs041200069. Date of registration: November 27, 2020.

**Supplementary Information:**

The online version contains supplementary material available at 10.1186/s12882-023-03277-0.

## Background

In recent decades, the number of patients with diabetes and prediabetes has increased worldwide [1] as well as in Japan [2]. Diabetes can damage major organs, including the kidneys, and the most serious consequence is end-stage renal disease, which requires renal replacement therapy [3]. A worldwide report revealed that the global annual incidence of end stage-renal disease (ESRD) among patients with diabetes has increased, but interestingly, in one region in Europe, the rate decreased [4]. Diabetic kidney disease (DKD) is the most common primary disease among prevalent and incident dialysis patients in Japan [5]. In addition, the presence of DKD markedly increases cardiovascular (CV) and mortality risk [6], which is why the prevention of DKD is a crucial global issue.

To delay the onset and progression of DKD, glycemic, blood pressure, and lipid control targets are set for diabetic patients; additionally, they are provided with advice on lifestyle changes, including nutritional management and exercise, to achieve their recommended targets [3]. Nevertheless, patients who cannot achieve the HbA1c target should be prescribed glycemic control drugs [3]. As sodium-glucose cotransporter 2 inhibitors (SGLT2i) are diabetic drugs that inhibit glucose reabsorption in the proximal renal tubule and promote urinary glucose excretion, they not only improve glycemic control independently of insulin-mediated mechanisms, but are also associated with body weight reduction [7]. Thus SGLT2i are thought to exert their glucose-lowering effect by reducing renal tubular glucose reabsorption, weight, systemic blood pressure, intraglomerular pressure, albuminuria, and slow GFR loss through mechanisms that appear to be independent of glycemia [7].

SGLT2i were first approved for use in 2012 in Europe and then in the US and Japan in 2014. Several SGLT2is are already widely used not only glycemic control but also for the reduction of CV mortality [8] and the incidence and exacerbation of DKD [9]. Although the renoprotective effect of SGLT2i is mainly thought to correct intraglomerular hypertension via restoration of tubular glomerular feedback [7], its mechanism is practically difficult to demonstrate. So far, few studies have attempted to elucidate the mechanisms in type 1 [10] and type 2 [11] diabetic patients; however, both trials enrolled patients with glomerular hyperfiltration. In this trial, we demonstrate how SGLT2i changes renal hemodynamics in DKD patients with moderate renal dysfunction. This renoprotective effect of canagliflozin derived from test of renal hemodynamics in diabetic kidney disease (FAGOTTO) study is expected to provide new evidence on the renoprotective effect of SGLT2i.

## Methods

### Trial design

The FAGOTTO trial is a multicenter, open-label, randomized (1:1), parallel-group study aimed at clarifying the effects of canagliflozin on renal hemodynamics and its safety in patients with type 2 diabetes and moderate DKD. This trial has been registered in the Japan Registry of Clinical Trials (jRCTs041200069). The study protocol was approved by the Certified Review Board of Nagoya University Graduate School of Medicine (No. CRB4180004). All patients will provide written informed consent to participate in this study after they receive information about the purpose of the study as well as the potential risks and benefits. Additional consent will be also confirmed for use of participant data and biological specimens in future ancillary study.

### Enrollment, randomization, and intervention

Participant recruitment has started in December 2020 at Nagoya University Hospital, Kasugai Municipal Hospital and Nagoya Kyoritsu Hospital in the Aichi Prefecture, Japan. The inclusion, exclusion, and termination criteria are listed in Table 1. Patients are being enrolled via a web-based registration and follow-up system organized by the Nagoya University Hospital’s Center for Advanced Medical and Clinical Research, Aichi, Japan. We are randomly allocating patients to two groups: the patients of canagliflozin (100 mg/day) group will receive the trial drug in the morning added to the patients’ usual treatment and the patients of control group will continue the conservative therapy. The allocation ratio is 1:1, and a dynamic allocation strategy using a minimization method is being used. The stratifying factors for randomization are eGFR (> 45 or ≤ 45 mL/min/1.73 m^2^), proteinuria (> 0.5 or ≤ 0.5 g/g Cr), age (> 70 or ≤ 70 years), HbA1c (> 7.0 or ≤ 7.0%), and the trial site.

**Table1:** Eligibility criteria

Inclusion criteria
**1.** Stable type 2 diabetes
**2.** eGFR (creatinine) ≤ 60 mL/min/1.73m^2^
**3.** Use of renin angiotensin system inhibitors (exclude intolerable or non-adaptive)
**4.** Age ≥ 18 years
**5.** Provision of written informed consent
Exclusion criteria
**1.** SGLT2 inhibitors administered within 8 weeks before providing informed consent
**2.** eGFR(creatinine) < 30 mL/min/1.73m^2^
**3.** Maintenance dialysis therapy or history of kidney plantation
**4.** Use of diuretics
**5.** Use of both ACEi and ARB (renin inhibitor)
**6.** Active nephritis or kidney disease
**7.** Signs of steroid diabetes
**8.** ALT and AST levels exceeding the facility standard value 2.0 fold
**9.** Heart failure NYHA class 4
**10.** Pregnant or planning pregnancy
**11.** Unsuitable for participation as assessed by attending investigator
Stop criteria
**1.** Withdrawal of consent
**2.** eGFR is dropping below 30 ml/min/1.73 m^2^ and medical decision to discontinue
**3.** Hypoglycemic symptoms and medical decision to discontinue
**4.** Ketoacidosis
**5.** Failure to attend hospital visits
**6.** Patient is found to be ineligible after starting per protocol treatment

### Intervention and control

Eligible participants will be randomly assigned (1:1) to the canagliflozin or control group. After a run-in period of up to 4–8 weeks, the canagliflozin group patients will be administered canagliflozin 100 mg orally once daily in the morning for 12 weeks. In contrast, in the control group, glucose levels will be controlled without any SGLT2i. Inulin and para-aminohippuric acid (PAH) clearance tests for the measurement of renal hemodynamics will be performed at baseline and 4 weeks after the start of the trial. A change in the type or dose of prior diabetic drugs and antihypertensive drugs is prohibited during the 12-week intervention period, but if side effects such as hypoglycemia and hypotension occur, reduction of the dose or discontinuation of these drugs is allowed. The use of the following drugs is restricted during the 12-week intervention period: diuretics, drugs that enhance the hypoglycemic effect, including β-blockers, salicylic acid agents, and monoamine oxidase inhibitors.

**Fig. 1:**
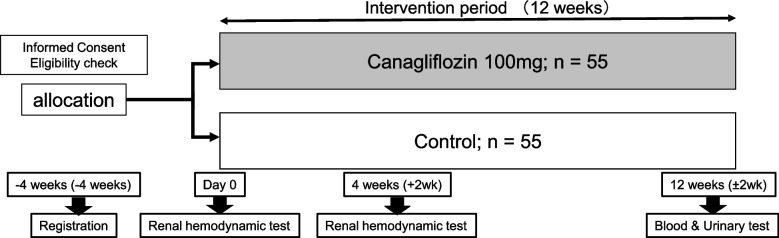
The study flowchart

The study flowchart is shown in Fig. 1.

### Renal hemodynamic calculation

mGFR and effective renal plasma flow (eRPF) will be calculated using inulin and PAH clearance, respectively. Briefly, 1% inulin and 0.5% PAH will be administered by means of a continuous intravenous infusion for 2 h under overnight fasting but hydrated conditions. If the participants are prescribed diabetic drugs and diuretics, they will be instructed to suspend the use of these drugs. Before infusion, participants will drink 500 ml of tap water in the outpatient treatment room to stimulate diuresis. During infusion, blood samples will be drawn 2 times at 45 and 105 min for measurement of serum levels of inulin and PAH, and urine samples will be collected between 45 and 105 min for measurement of urinary inulin and PAH excretion after completely emptying the bladder 45 min after the start of the infusion.

The filtration fraction (FF), effective renal blood flow (eRBF), and renal vascular resistance (RVR) will be calculated by dividing mGFR by eRPF, eRPF by (1-hematocrit), and mean blood pressure by eRBF, respectively. eGFR from creatinine levels (eGFRcreat) will be calculated as follows: eGFRcreat (mL/min/1.73 m^2^) = 194 × SCr^−1.094^ × Age^−0.287^ (× 0.739: coefficient for female) [12].

### Data collection and other determinant

Baseline characteristics, medication information, vital signs, and biochemical data will be collected. Body weight will be measured at arrival in the clinic (before the inulin clearance test) and blood pressure will be measured twice in the sitting position before and after the inulin clearance test. Measurements of blood urea nitrogen, creatinine, hematocrit, HbA1c, alanine aminotransferase (ALT), aspartate aminotransferase (AST), proteinuria, and albuminuria are planned at baseline, 4–6 weeks after the start of the trial, and at the end of the trial and will be performed using routine procedures at the clinical chemistry facilities of each hospital. Serum and urinary inulin levels will be measured in the laboratory of SRL Inc. (Aichi, Japan). Serum and urinary PAH levels will be measured using liquid chromatography mass spectrometry (LC–MS/MS) at the Institute of Transformative Bio-Molecules (ITbM), Nagoya University, Aichi, Japan. Urinary angiotensinogen levels will be measured using a sandwich ELISA system at the Department of Pharmacology, Kagawa University, Kagawa, Japan. For quality control and quality assurance, a data monitoring committee is composed and monitoring adherence to the protocol. Auditing trial conduct is scheduled and the process will be independent from investigators and the sponsor.

### Endpoints

The primary endpoint of the protocol is the percentage change in FF after 4 weeks of treatment in the canagliflozin and control groups. The secondary endpoints are (1) the change in FF, percentage change, and change in (2) mGFR, (3) eRPF, (4) eRBF, and (5) RVR after 4 weeks of treatment; (6) changes in eGFR, HbA1c, and albuminuria after 4 and 12 weeks of treatment; and (7) trend of blood pressure and body weight after 4 and 12 weeks of treatment in the canagliflozin and control groups. In addition, we plan to (8) compare the changes in urinary angiotensinogen levels between the canagliflozin and control groups after 4 weeks of treatment. Moreover, the rate of adverse drug reactions will be assessed for safety.

### Sample size

For the purpose of comparing independent results, we decided to use power of 0.85 and a significance level of 0.05, to detect a significant difference. The difference in the percentage change in FF after 4 weeks of treatment in the canagliflozin group compared with that in control group was assumed to be 60% of the standard deviation. Based on these calculations, we determined that at least 50 individuals are necessary for each group. Taking into account potential discontinuation and withdrawal, we set the target number for the canagliflozin group to 55, with 55 control patients, and thus, 110 patients were planned to be included in total. Additionally, we took into account the actual number of patients who met the registration criteria at each facility.

### Statistical analyses

Validity/safety will be analyzed in the largest analysis target group (full analysis set; FAS), defined below. To be included in the FAS, subjects must satisfy these two criteria: (1) meet the selection criteria and not meet the exclusion criteria, and (2) have at least one marker to evaluate efficacy after administration (regardless of the primary or secondary endpoint). The primary aim of the analysis is to compare the percentage changes between the canagliflozin group and the control group at 4 weeks after administration using analysis of covariance (ANCOVA) with eGFR, proteinuria, age, and HbA1c as covariates. No interim analysis is planned.

The analysis methods for secondary outcomes are shown in Table 2.

**Table 2:** Analysis methods for secondary outcomes of the canagliflozin and control groups

Secondary outcomes	The analysis methods
1. Percentage change and the change in eRPF, eRBF, and RVR after 4 weeks of treatment	• Compare the percentage changes between the canagliflozin group and the control group at 4 weeks after administration by analysis of covariance (ANCOVA) with eGFR, proteinuria, age and HbA1c as covariates
2. Change in FF, eRPF, eRBF, and RVR after 4 weeks of treatment	• Compare the percentage changes between the canagliflozin group and the control group at 4 weeks after administration by analysis of covariance (ANCOVA) with FF, eRPF, eRBF, and RVR at the baseline and eGFR, proteinuria, age, and HbA1c as covariates
3. Changes in eGFR, HbA1c, albuminuria after 4 weeks and 12 weeks of treatment, trend of blood pressure and body weight after 4 weeks and 12 weeks of treatment, and change in urinary angiotensinogen levels after 4 weeks of treatment	• Calculate the adjusted mean and the 95% CI for changes at each time point by a linear mixed model with each treatment group, time point, and interaction of the treatment group and time point as the fixed effects • Compare between treatment groups using a linear mixed model with values of each index at baseline, treatment groups, time points, interaction between treatment groups and time points as the fixed effects and changes of each index as response variables • Compare changes of each index among treatment groups at each time point using the Tukey–Kramer method to correct for multiplicity
4. Rate of adverse drug reactions	• Calculate the 95% confidence interval for each treatment group and compare it between the groups using χ2 test (or Fisher's exact test)

*ANCOVA* Analysis of covariance, *FF* Filtration fraction, *eRPF* Effective renal plasma flow, *eRBF* Effective renal blood flow, *RVR* Renal vascular resistance

### Dissemination

Nagoya University Graduate School of Medicine will submit results of this study to a peer-reviewed journal on the basis of own judgment from a scientific and neutral standpoint with complying confidentiality obligations.

## Discussion

Several large-scale randomized control studies have provided evidence of the cardioprotective and renoprotective effects of SGLT2i [13]. Among them, a randomized clinical trial of empagliflozin was the seminal study. The trial was designed to assess the cardioprotective effect of empagliflozin in type 2 diabetic patients with > 7.0% of HbA1c and a high risk of CV events [14]. It demonstrated that patients who received empagliflozin had lower rates of hospitalization for heart failure as well as CV and all-cause mortality [8]. The authors also demonstrated renoprotective effects as the secondary microvascular outcome of this trial included a significant relative risk reduction of doubling of serum creatinine level and initiation of renal replacement therapy in patients whose eGFR was at least 30 ml/min/1.73 m^2^ [9]. Notably, eGFR of the empagliflozin group showed a short-term decrease in the first 4 weeks and remained stable from long-term administration to the end of the trial, while that of the placebo group declined steadily [9]. Next, a convincing report from the integrated CANVAS program showed that canagliflozin prevented the progression of albuminuria and renal events; specifically, findings showed a 40% reduction in eGFR, renal replacement therapy, or renal death in type 2 diabetic patients with inadequate glycemic control and an elevated risk of CV diseases with a mean eGFR of approximately 76 ml/min/1.73m^2^ [15, 16]. There was the CREDENCE trial designed primarily to assess the renoprotective effect of canagliflozin in patients with type 2 diabetes [17]. In this trial, the inclusion criteria specified patients with type 2 diabetes with over 6.5% of HbA1c and risk factors for the progression of kidney disease, such as albuminuria, who were treated with renin-angiotensin system blockade; enrollment included approximately 60% patients with stage 3 CKD [17]. Although the subjects in the CREDENCE trial had low eGFR at the start in contrast to those included in the previous CV outcome trials of SGLT2i [9, 16], canagliflozin showed similar effects, including a lower risk of end-stage renal disease, doubling of serum creatinine level, or death from renal causes, compared to placebo [18]. Based on these clinical trials, the renoprotective effect of SGLT2i is now recognized, but the magnitude of its effectiveness in individual patients remains unclear. A systematic review reported that the renoprotective effect of SGLT2i was greater in diabetic patients with preserved renal function at baseline than in those with moderate CKD, whereas the cardioprotective effect was greater in patients with poorer renal function [13]. Although an eGFR dip is observed in the eGFR curve after prescribing SGLT2i, a recent editorial indicated that a larger dip generally resulted in a stronger benefit of SGLT2i than placebo [19]. Thus, to demonstrate whether SGLT2i can modulate renal hemodynamics even in patients at high risk of end-stage renal disease, our trial will evaluate renal hemodynamics during an eGFR dip in patients with moderate renal dysfunction who are administered SGLT2i.

The possible renal protective effects of SGLT2i are diverse, including lowering blood pressure via natriuresis and osmotic diuresis, reducing hypoxia in the proximal tubule via tubular workload reduction, and anti-inflammation-fibrosis action [20–22]. Among these actions of SGLT2i, correction of intraglomerular hypertension via restoration of tubular glomerular feedback is thought to be the most crucial and dynamic [21]. An experimental study in an animal model of type 1 diabetes demonstrated that SGLT2i induced afferent arteriolar vasoconstriction and decreased the single-nephron glomerular filtration rate via the tubule-glomerular feedback mechanism that resulted in the reduction of intraglomerular pressure, using in vivo multiphoton microscopy imaging techniques [23]. Cherney et al. demonstrated that SGLT2i attenuated glomerular hyperfiltration in patients with type 1 diabetes mellitus with glomerular hyperfiltration defined by eGFR ≥ 135 mL/min/1.73 m^2^; attenuation of glomerular hyperfiltration was accompanied by a decrease in renal blood flow and an increase in renal vascular resistance [10]. The authors discussed that such a reduction of glomerular hyperfiltration was mediated by preglomerular vasoconstriction by affecting the tubular-glomerular feedback mechanism, and long-term use of SGLT2i might reduce intraglomerular pressure, resulting in an overt renoprotective effect [10]. Interestingly, this trial failed to show this evidence in patients with normal glomerular filtration [10]. Recently, van Bommel et al. conducted a trial to evaluate the renal hemodynamic effect of SGLT2i in patients with type 2 diabetes mellitus with eGFR ≥ 60 mL/min/1.73m^2^ treated with metformin monotherapy [11]. They showed that SGLT2i lowered the mGFR without increasing renal vascular resistance, and these effects were accompanied by postglomerular vasodilation [11]. Interestingly, the ability to lower postglomerular resistance was independent of blood glucose concentration [11]. Although clinical studies have demonstrated a renoprotective effect of SGLT2i in patients with type 2 diabetes who have moderate renal dysfunction, as discussed above, changes in renal hemodynamics after SGLT2i administration in these patients are still unclear. This trial will contribute to research in the field by elucidating the renal hemodynamic mechanism of SGLT2i and, provide a significant basis for the prescription of SGLT2i in patients with DKD if we demonstrate its ability to control intraglomerular pressure in our subjects.

The target number of patients is 110 (1:1 = canagliflozin group: control group). This protocol was approved by the Certified Review Board of the Nagoya University Graduate School of Medicine on November 6, 2020 (Version 1.2), and opened to the public on the website of the Japan Registry of Clinical Trials on November 27, 2020. We enrolled the first patient on December 23, 2020. By the end of June 2022, we enrolled 81 patients from three participating hospitals and further recruitment is ongoing. This study is expected to end in 2025. The FAGOTTO study is the first randomized controlled study of canagliflozin to assess renal hemodynamics in patients with type 2 diabetes and moderate renal dysfunction. Although further studies will be needed, the data from this study will provide evidence for optimal therapy to prevent progression of DKD.

### Supplementary Information

**Additional file 1. **SPIRIT Checklist.

**Additional file 2. **WHO trial registration data set.

## Data Availability

Not applicable.

## Abbreviations

ALT: Alanine aminotransferase
ANCOVA: Analysis of covariance
AST: Aspartate aminotransferase
CVD: Chronic kidney disease
CV: Cardiovascular
DKD: Diabetic kidney disease
ESRD: End stage-renal disease
FF: Filtration fraction
GFR: Glomerular filtration rate
PAH: Para-aminohippuric acid
RBF: Renal blood flow
RPF: Renal plasma flow
SGLT2i: Sodium-glucose cotransporter 2 inhibitors
