# Supplementary material for: Rationale and study design of a randomized controlled trial to investigate the renoprotective effect of canagliflozin assessed by test of renal hemodynamics in diabetic kidney disease (the FAGOTTO study)
Source: BMC Nephrol. 2023 Aug 3;24:228. doi: 10.1186/s12882-023-03277-0 (PMC10401745; doi:10.1186/s12882-023-03277-0)
Supplement: Supplementary file 2 — Additional file 2. WHO trial registration data set. [file 12882_2023_3277_MOESM2_ESM.docx]

WHO trial registration data set

| Primary Registry and Trial Identifying Number | jRCTs041200069 |
| --- | --- |
| Date of Registration in Primary Registry | 7 April 2022 |
| Secondary Identifying Numbers | N/A |
| Source(s) of Monetary or Material Support | Mitsubishi Tanabe Pharma Corporation |
| Primary Sponsor | Nagoya University Graduate School of Medicine, Japan |
| Secondary Sponsor(s) | N/A |
| Contact for Public Queries | Dr. Sawako Kato  kato07@med.nagoya-u.ac.jp |
| Contact for Scientific Queries | Prof. dr. Shoichi Maruyama  marus@med.nagoya-u.ac.jp |
| Public Title | FAGOTTO study |
| Scientific Title | Renoprotective effect of canagliflozin derived from test of renal hemodynamics in diabetic kidney disease |
| Countries of Recruitment | Japan |
| Health Condition(s) or Problem(s) Studied | Diabetes Mellitus, Type 2 |
| Intervention(s) | arm A ; CANAGLU tablet 100mg /day  arm B ; control |
| Key Inclusion and Exclusion Criteria | *Table 1 |
| Study Type | A multicenter, open-label, randomized (1:1), parallel-group study |
| Date of First Enrollment | 23 December 2020 |
| Recruitment Status | Recruiting |
| Primary Outcome(s) | *Methods, endpoint |
| Key Secondary Outcomes | *Methods, endpoint |
| Ethics Review | the Certified Review Board of Nagoya University Graduate School of Medicine (No. CRB4180004) |
| Completion date | N /A |
| Summary Results | N /A |
| IPD sharing statement | No |
